# Cracking the seed code: How nitrate directly breaks ABA signaling for germination

**DOI:** 10.1093/plcell/koaf072

**Published:** 2025-06-13

**Authors:** Nitin Uttam Kamble

**Affiliations:** Assistant Features Editor, The Plant Cell, American Society of Plant Biologists; Indian Institute of Science Education and Research, Thiruvananthapuram 695551, India

Have you ever wondered how seeds can be stored for years and still give rise to the next generation of life? Extensive research has shown that the plant hormone abscisic acid (ABA) plays a crucial role in maintaining seed dormancy and desiccation tolerance (reviewed by Sajeev et al. 2024). ABA regulates this process through intricate molecular mechanisms involving the ABA receptor complex (PYR/PYL/RCAR), protein phosphatases (PP2Cs), and kinases (SnRK2s), along with ABA signaling pathway transcription factors (TFs) such as ABSCISIC ACID INSENSITIVE 3 (ABI3) and ABI5 (Lopez-Molina et al. 2001).

Interestingly, nitrogen not only determines plant growth and yield potential as a macronutrient but also acts as a key signaling molecule. Plants primarily take up nitrogen from the soil in the form of nitrate (NO_3_^−^), which, upon reduction to nitrite, releases nitric oxide (NO). Both nitrate and NO have been shown to stimulate seed germination. NO functions through PROTEOLYSIS6 (PRT6) to negatively regulate *ABI5* transcription and induce ABI5 *S*-nitrosylation, leading to its degradation and modulation of the ABA response (Gibbs et al. 2014; Albertos et al. 2015). Despite this knowledge, whether nitrate serves as a crucial cue that directly regulates ABA signaling during seed germination remains unknown.

In new work, **Zhichong Huang and colleagues (Huang et al. 2025)** explore the role of nitrate in ABA-regulated seed germination. Huang et al. first demonstrated that nitrate mitigates the inhibitory effects of ABA during Arabidopsis (*A. thaliana*) wild-type Columbia seed germination. Seeds germinated on water agar supplemented with ABA and KNO_3_ exhibited better germination and cotyledon greening compared with controls germinated on media containing ABA alone. Using double mutants of NITRATE REDUCTASE1 (NIA1) and NIA2 (*nia1_nia2*), which are impaired in NO synthesis, along with *prt6* (a NO perception mutant), the authors observed an improvement in germination when both ABA and KNO_3_ were present, as opposed to when only ABA was present, demonstrating that nitrate modulates ABA signaling independently of NO signaling.

Huang et al. further studied if nitrate can specifically modulate ABI3 and ABI5 functions as these TFs are well studied in ABA-repressed seed germination. They found that *ABI3* and *ABI5* transcript levels were lower in seeds germinated on ABA + KNO_3_ compared with those on ABA with or without KCl. In addition, KNO_3_, but not KCl, partially suppressed the ABA hypersensitivity of *ABI3* and *ABI5* overexpression seeds.

Huang et al. then focused on NIN-LIKE PROTEIN (NLP) TFs known to play key roles in nitrate-mediated gene expression (Figure). Through protein–protein interaction studies, they confirmed that NLP8 physically interacts with ABI3 and ABI5 both in vitro and in vivo. This interaction was responsive to both ABA and nitrate, with strong interaction observed with the phosphorylated form of ABI5. Notably, NLP8 is highly abundant in mature dry and imbibed seeds and is induced by ABA but not by nitrate. Importantly, only NLP8, and not NLP7 or NLP9, could suppress ABA signaling and promote seed germination despite all 3 showing interaction with ABI3 and ABI5, suggesting that nitrate inhibits ABA signaling specifically through NLP8.

**Figure.**
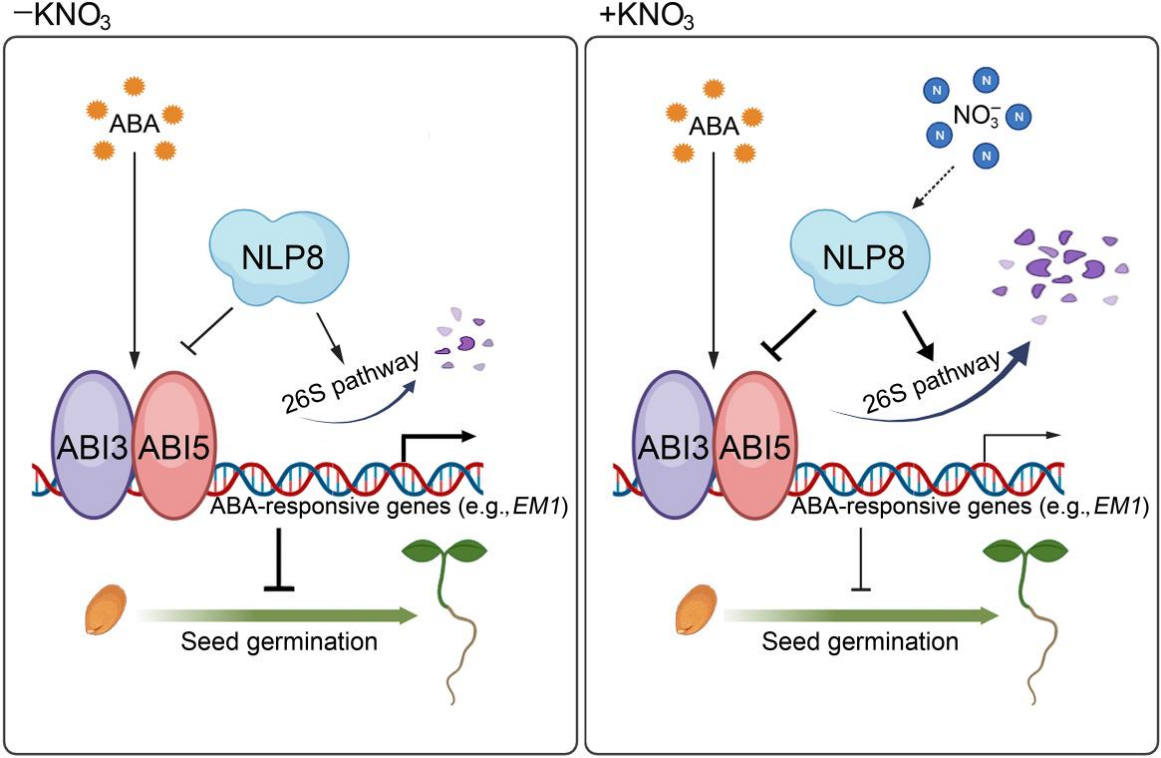
ABI3 and ABI5 suppress seed germination in an ABA-dependent pathway. ABI3 and ABI5 physically interact with the nitrate-related protein NLP8 in a nitrate- and ABA-responsive manner. NLP8 interferes with the transcriptional functions of ABI3 and ABI5, negatively regulating ABA signaling during seed germination. Reprinted from Huang et al. (2025), Figure 10.

Through comprehensive double and triple mutant analyses, the authors demonstrated that NLP8 functions upstream of ABI3 and ABI5 in modulating ABA signaling, as the ABA hypersensitivity of *NLP8* mutants required functional ABI3 and ABI5 (Figure). Additional protein DNA interaction studies showed that NLP8 attenuates ABI3 and ABI5 function through protein–protein interactions, thereby modulating the expression of downstream target genes such as *LATE EMBRYOGENESIS ABUNDANT1* (*EM1*) and *EM6*. Additionally, they found that nitrate and NLP8 promote the degradation of ABI3 and ABI5 via the 26S proteasome pathway, reducing their suppressive effects on seed germination and early seedling establishment (Figure).

In summary, these findings reveal that nitrate attenuates the ABA signaling pathway independently of NO signaling through NLP8. Specifically, NLP8 directly interacts with ABI3 and ABI5 to regulate downstream gene expression. The coordination between the nitrate and ABA signal transduction pathways guarantees that ABA signaling remains regulated at an optimal level under nitrate-sufficient conditions. Nevertheless, the precise mechanisms by which nitrate promotes NLP8 association with and degradation of ABI3/ABI5 via the 26S proteasome pathway remain to be unraveled. Does the increased interaction following nitrate or ABA pretreatment arise from a similar mechanism, and is nitrate binding by NLP8 relevant in the context of nitrate pretreatment? Understanding these processes could have implications for improving seed germination under diverse physiological conditions.

## Recent related articles in *The Plant Cell*

- Du et al. (2024) report on negative regulation of ABA signaling by AFP1-mediated degradation of ABI5 through the U-box E3 ubiquitin ligase PUB35.
- Durand et al. (2024) report on NLP2 as a top-tier transcriptional regulator of the early nitrate response gene regulatory network.
- Mei et al. (2023) report that Auxin contributes to jasmonate-mediated regulation of abscisic acid signaling during seed germination in Arabidopsis.
- Shanks et al. (2024) review research that uses systems biology approaches to examine temporal and spatial mechanisms behind nitrogen sensing and signaling in plants.
- Zhao et al. (2024) report on effect of *S*-nitrosylation of the transcription factor MYB30 in NO-promoted seed germination in Arabidopsis.

## Dive Curated Terms

The following phenotypic, genotypic, and functional terms are of significance to the work described in this paper:

ABI5 Gramene: AT2G36270
ABI5 Araport: AT2G36270

ABI3 Gramene: AT3G24650
ABI3 Araport: AT3G24650

MYB30 Gramene: AT3G28910
MYB30 Araport: AT3G28910

PRT6 Gramene: At5g02310
PRT6 Araport: At5g02310

NLP7 Gramene: AT4G24020
NLP7 Araport: AT4G24020

PUB35 Gramene: AT4G25160
PUB35 Araport: AT4G25160

NLP8 Gramene: AT1G20640
NLP8 Araport: AT1G20640
